# What information do parents need when facing end-of-life decisions for their child? A meta-synthesis of parental feedback

**DOI:** 10.1186/s12904-015-0024-0

**Published:** 2015-04-30

**Authors:** Vicki Xafis, Dominic Wilkinson, Jane Sullivan

**Affiliations:** 1 Discipline of Obstetrics and Gynaecology, The University of Adelaide, Adelaide, Australia; 2 John Radcliffe Hospital Oxford, Director of Medical Ethics, Oxford Uehiro Centre for Practical Ethics, University of Oxford, Oxford, UK; 3 Children’s Bioethics Centre, The Royal Children’s Hospital, Melbourne, Australia; 4 The Centre for Health Equity, The University of Melbourne, Melbourne, Australia

**Keywords:** End of life care, Consumer health information, Withholding treatment, Intensive care, Decision making

## Abstract

**Background:**

The information needs of parents facing end-of-life decisions for their child are complex due to the wide-ranging dimensions within which such significant events unfold. While parents acknowledge that healthcare professionals are their main source of information, they also turn to a variety of additional sources of written information in an attempt to source facts, discover solutions, and find hope.

Much has been written about the needs of parents faced with end-of-life decisions for their child but little is known about the written information needs such parents have. Research in the adult intensive care context has shown that written resources impact positively on the understanding of medical facts, including diagnoses and prognoses, communication between families and healthcare professionals, and the emotional wellbeing of families after their relative’s death.

**Methods:**

A meta-synthesis of predominantly empirical research pertaining to features which assist or impede parental end-of-life decisions was undertaken to provide insight and guidance in our development of written resources (short print and online comprehensive version) for parents.

**Results:**

The most prominently cited needs in the literature related to numerous aspects of information provision; the quantity, quality, delivery, and timing of information and its provision impacted not only on parents’ ability to make end-of-life decisions but also on their emotional wellbeing. The meta-synthesis supports the value of written materials, as these provide guidance for both parents and healthcare professionals in pertinent content areas.

**Conclusions:**

Further research is required to determine the impact that written resources have on parental end-of-life decision-making and on parents’ wellbeing during and after their experience and time in the hospital environment.

**Electronic supplementary material:**

The online version of this article (doi:10.1186/s12904-015-0024-0) contains supplementary material, which is available to authorized users.

## Background

Although most children and newborn infants who are admitted to intensive care survive, a small proportion (3-6%) does not [1,2]. For many of these children (between 75 and 90% of infants who die in intensive care units), death is preceded by a discussion between parents and the medical staff, and by an explicit decision to limit treatment that could potentially have prolonged life (this is referred to as an “end-of-life decision”) [2-6]. Where the newborn infant or child’s prognosis is very poor, these decisions are widely regarded as not only legally acceptable, but as ethically good practice, since they prevent suffering and the prolongation of the child’s death [7-10].

Provision of written information has been used to improve communication and outcome for families in adult intensive care. A family information leaflet, provided at the time of admission to intensive care, reduced the proportion of family members with poor understanding of diagnosis and prognosis from 40% to 11% [p < 0.0001] [11]. Similarly, written information in a brochure on bereavement provided to the families of dying patients (in conjunction with a modified family conference), was associated with reduced anxiety, depression and post-traumatic stress symptoms 3 months later [12].

Written information might, thus, provide important support for parents making end-of-life decisions for their child. However, there is a need to determine what information parents require, and consequently what such written material should contain. Previous published reviews have focused on factors that enable parental input into decisions [13], the evaluation of levels of involvement in decision-making [14,15], parental use of information in the illness trajectory [16], and parental needs in neonatal intensive care units [17]. None, however, have considered the written information needs of parents facing end-of-life decisions for their child. In addition, there has been no attempt to synthesize the findings of related studies for the purpose of identifying parents’ written information needs.

## Methods

### Aim of meta-synthesis

A hermeneutic approach was adopted in this work to inform the development of a handbook and online resource for parents facing end-of-life decisions for their very ill or dying children [18,19]. A preliminary search revealed a dearth of literature specifically focusing on written information for parents making end-of-life decisions for their child. Therefore, we sought to identify issues that parents have identified as being central to their ability to make decisions in the context of end-of-life decision-making, which might provide insight into parents’ written information needs. Specifically, we aimed to identify communication or information-related features that parents report as being helpful or unhelpful during end-of-life decision-making. We ultimately aimed to incorporate the communication and information related features of the meta-synthesis findings in the written resources we developed.

### Design

We conducted the literature search in August-November 2012, searching the bibliographic database Scopus (search terms were: (perinatal OR newborn OR neonat* OR preterm OR premature OR infant*) AND (palliative OR “end of life” OR withhold* OR withdrawal*) AND (attitude* OR view* OR perception*) AND (parent* OR mother* OR maternal OR father* OR paternal OR families OR family)). Scopus was used as its data sources include a number of resources such as MEDLINE, EMBASE, open access papers, as well as grey literature [20].

We included studies relating to critically ill children and newborn infants published between 1990 and 2012 that addressed at least one of the inclusion criteria below:

1. What unmet needs do parents identify in the decision-making process?
2. What features do parents find helpful or alternatively unhelpful/damaging in the decision-making process?
3. Which resources have parents found helpful?
4. What suggestions are put forward by parents for communication?

**Figure 1:**
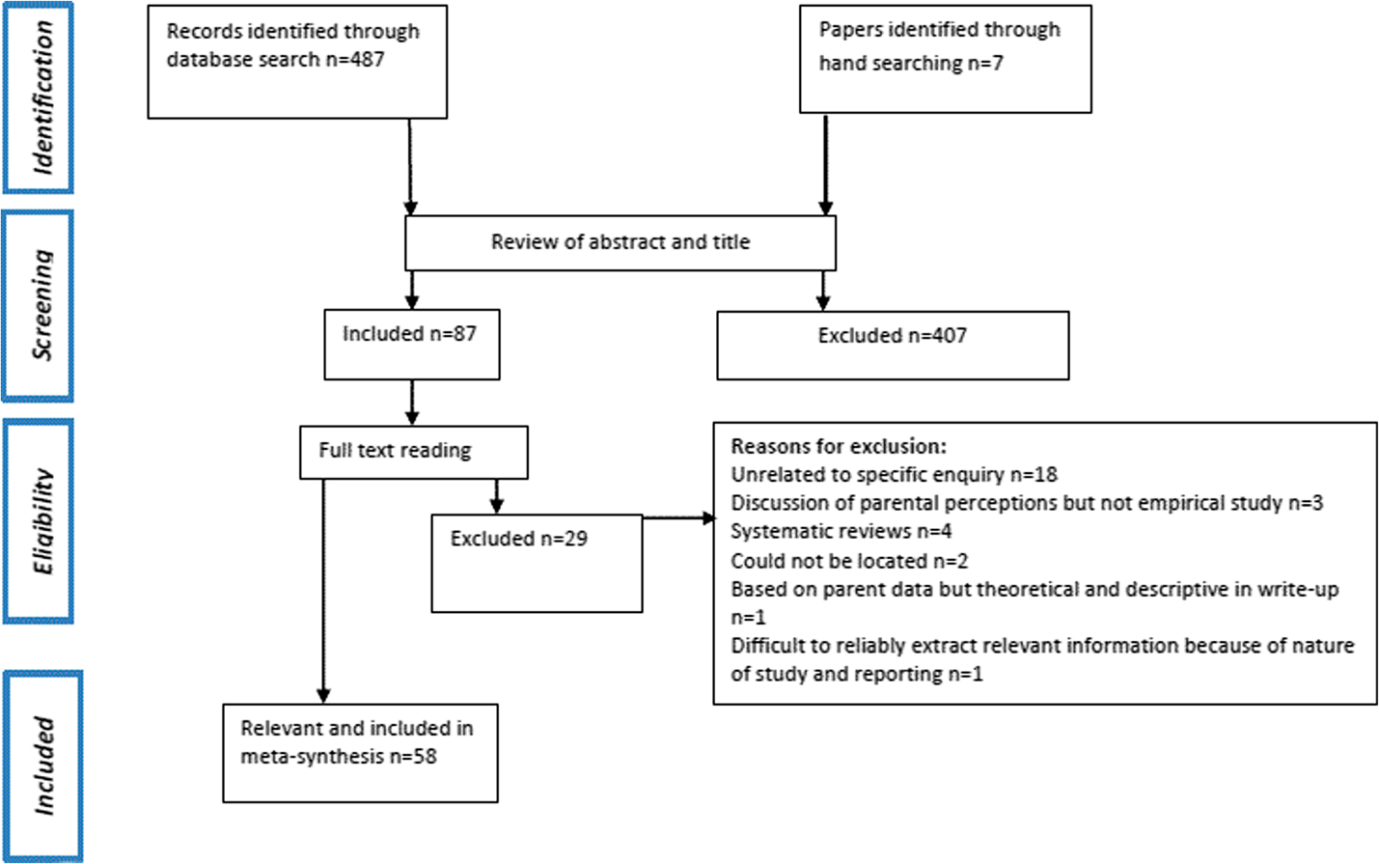
Flow of studies. Provides information on the identification, selection, and inclusion of the studies reviewed.

The initial search produced 487 publications (see Figure 1). Additional papers (n=7) were identified by hand searching the personal libraries of the authors, and reference lists of relevant articles. Following review of the title and abstract 87 were retrieved and read in full text. After the papers were scanned for relevance, the publications were further reduced to 58 for inclusion in the meta-synthesis (see Additional file 1: Table S1). The first author conducted the review of papers.

The studies included were in the vast majority qualitative. However, some studies comprised both a qualitative and quantitative component and a very small number based on closed survey responses from parents were also included because of the relevance of their content. The main focus was on studies that involved more than one parent/family but personal accounts were also included. Studies in languages other than English were not included.

As the review was primarily one of qualitative research, the qualitative research designs employed in the studies varied significantly. For example, some used an ethnographic approach, others phenomenology, and still others were case studies (see Additional file 1: Table S1). Due to the nature and intent of the meta-synthesis, no restrictions relating to study design or scientific merit were imposed on the studies identified so as not to exclude data of relevance to the purpose of the meta-synthesis [21]. The findings of the studies were not formally appraised. We were particularly interested in the sections of the papers that revealed parental views relating to communication and/or information that aids or creates difficulties in decision-making rather than the overall findings of the studies. Therefore, appraising the overall findings of the studies, which were often unrelated specifically to communication and/or information that aids or creates difficulties in decision-making, was not relevant to the purpose of the search, as this work was not a systematic review of the literature, but rather, specifically focused on studies that included any information relevant to our topic of interest.

In conducting the review of the literature, it was recognised that there may be great variability in parents’ perceptions and that the views of a single parent were equally important as those of a group of parents, as individuals have different needs depending on a complex variety of personal, social, and cultural factors.

### Meta-synthesis of findings

All relevant studies were electronically searched for sections relating specifically to communication and/or information and end-of-life decision-making, which addressed at least one of the selection criteria. These sections were extracted and included in an Excel spreadsheet by the first author. Descriptive codes were assigned to sections based closely on the inclusion criteria (unmet needs, helpful features, unhelpful features, resources, suggestions). All studies were coded deductively into pre-determined concepts considered and agreed upon at the outset of the review. Relevant sections were highlighted, coded, and compared and contrasted. The codes were subsequently re-examined and the content areas expressed in the selection criteria served as coding frames under which the codes were sorted. A new content area pertaining to *factors influencing decision-making* emerged*.* These were factors identified by parents who had participated in the studies reviewed as impacting either on their ability to make decisions or on the kinds of decisions made. Constant in the meta-synthesis was a consideration of the oftentimes specific circumstances surrounding parents’ comments and care was taken not to alter the nature of the contributions made by parents by reporting on them more generally or specifically than the contributors had intended when participating in the respective studies. The importance of preserving the original meaning in such meta-syntheses is highlighted in Walsh and Downe [22].

### Ethics

Ethics approval was not required for this work, as it was a meta-synthesis of published literature and did not involve the collection of data from research participants. Consequently, written informed consent was not sought from anyone.

## Results

Studies generally targeted parents but some focused exclusively on mothers’ views therefore perhaps resulting in an under-representation of fathers’ views. Parents identified factors, barriers, unmet needs, and areas requiring attention. Themes were retained in the areas under which they were identified, but overlapped between sections of the meta-synthesis. We first review some of the recurring issues influencing decision-making raised by parents and later comment on their import in relation to written materials.

### Key factors influencing decision-making

Parents’ grief, their powerful emotions, the intensive care unit environment, and the nature of the decisions they faced were reported to affect information processing and parents’ ability to make decisions [23-26]. Parents reported that their decision-making capacity was reduced by a paucity of information and that their comprehension of information was hampered by medical jargon used [26] and their difficulties in understanding and retaining complex explanations about their child’s condition [27,28].

“You read books and even attend classes during pregnancy to help prepare to have a baby, even though that’s ‘normal’. It’s horrendous, but even more important to have some help to prepare yourself for losing your precious baby. Mostly because that doesn’t feel ‘normal’ at all and you haven’t a clue what to do” [23].

“It might be immoral to push you to make a decision…you were too tired to make. Maybe six months later there is a kind of sobering-up process. Maybe if she were still in [the] situation, critical, being sustained like that, with no hope, or little hope, I would start to think differently” [28].

Interviews with parents revealed that potential disability in their child was an important consideration for some parents, particularly when parents had first-hand knowledge of people who suffered severe disabilities [29]. Such considerations, however, appear to differ depending on the parents’ country of origin [30]. Consideration of the child’s quality of life ranked high as an influencing factor in the decisions parents ultimately made [31-33], as did a desire to reduce the child’s pain and suffering [31-34].

Withdrawal of treatment was not considered appropriate by all parents [33], but some parents accepted that a decision to withdraw treatment was the right decision, especially when they felt that no other treatment options were available [35]. Many parents described feeling conflicted; for example, in one study parents described being torn between what they thought was in the best interest of their child and what they, as parents, wanted [25]. The conflict between these opposing needs caused them to face extreme emotional strain when they had to make decisions about withdrawal of nutrition. Parents of older children recognised that their needs and interests were subordinate to their children’s [32].

Medical prognoses did not always weigh heavily in parents’ decisions [33,36]. A relative lack of regard for medical prognoses appeared to be related to parents’ lack of ability to comprehend information imparted, parents feeling emotionally depleted, and mothers being confronted by their own medical conditions [36]. For some mothers, factors influencing decision-making included personal values, and beliefs or experiences rather than medical facts [37]. Finally, other key factors influencing parental decision-making included hope [34,36], religion [32,38-40], and spirituality [36]. For example, mothers reported that they could not trust doctors who showed no hope for a better outcome and who appeared to have abandoned efforts for a better outcome [36].

“…It’s really important for a parent to hear some hope, although the rationale says that this is 90% going to happen this way negatively” [36].

### What unmet needs do parents identify in the decision-making process?

Most parents had never faced end-of-life decisions before. Parents expressed the view that they were in completely unknown territory and needed some insight into what to expect [23]. In several studies, parents lamented the fact that the treating doctor had not provided more information [34,41,42]; some parents felt that a lack of information had impeded their ability to make decisions [27].

“They don’t tell parents until the parents show concern [… or if] there is a chance of not getting the severity level right […] I think that is wrong. Even if they only slightly think there might be a problem, they should be right on it, explaining it and giving you a list of things you can do. [Not knowing what to do] feels terrible” [42].

Access to healthcare professionals was also raised as being limited, an issue which impeded access to information for parents [43]. In one study, parents reported a lack of information about the condition of their child as well as information about stopping or starting treatments [41].

Parents expressed a need to know more about the intensive care unit and contextual information about the environment [24]. This included the language of intensive care and treatment. Medical jargon was described as confusing and overwhelming for parents [24,27,36,43-45], especially if English was not parents’ first language [44,46], and some parents recognised that an understanding of clinical information was acquired over time [42].

“I kept asking, ‘What is this? What are you telling me you are going to do for her?’ They gave me answers in medical terminology. This is what I kept getting, and I’m like, ‘Could you explain that?’ No one really explained it to my satisfaction because I did not and still do not understand. And I would like to understand it in layman’s terms. It was what you were gonna do for her” [43].

Parents sometimes lacked information about supports that might be available. For example, some parents had not been advised of hospital interpreters’ services [44]. Several parents regretted that they had not had the opportunity to discuss their situation with another parent who had been through a similar experience [47,48]. One mother believed that such contact would have better prepared her for the ensuing events and would have had a positive effect on how she dealt with the situation emotionally [47]. Some parents expressed the view that an advocate would have assisted them in asking questions of healthcare professionals as well as in making sense of information received [42].

Parents expressed a need for culturally sensitive and relevant information. Some couples faced additional difficulties, as a result of religious requirements. For example, doctors caring for one Muslim mother were unable to understand why the mother was so anxious about her baby drawing breath [49], not realising that unless the baby breathed, he/she would not be recognised as being Muslim, which would have great implications for the child and the child’s parents.

### What information do parents find helpful in the decision-making process?

The information included in this section is important to the development of any written information for parents, as it relates to ways of communicating information that assist parents. Such nuanced understanding of parental needs impacts on the style, the layout, the content, the tone, the language, and the stance adopted in the development of written resources aimed at assisting parents in making end-of-life decisions.

Parents indicated that an understanding of the situation and an appreciation of what to expect, irrespective of the end result, was important in preparing them for the outcomes [23]. These parents felt empowered through the provision of accurate information delivered in a timely and sensitive manner [23]. Likewise, parents in another study indicated that they wanted easy access to information that was not ‘sugar coated’ and felt that such information was truthful [50]. The provision of information was linked to a sense of control over fathers’ experience in the neonatal intensive care unit and was regarded as central to their ability to make decisions even though fathers expressed the need for varying amounts of information, with some admitting that too much information was confronting [51]. Similarly, parents in another study felt they were more in control and less fearful when information was uncomplicated, candid, and delivered in simple terms [52].

“Give us that knowledge you know, educate us so we can have some answers. We had to ask for his CAT scan….Obviously we are not medical students and a lot of the stuff may be you know a little tough to understand, but it can be broken down. We will comprehend it if you just lay it out there” [50].

When the time came for funeral arrangements, these same parents, who had not had previous experience with the death of a baby, found that the guidance and information they were given, as well as the increased amount of time they required were all helpful factors in coming to decisions [52]. Carefully and clearly formulated explanations assisted parental decision-making, as did written accounts of the advantages and disadvantages of the options medical professionals had discussed with parents [53]. Comprehensive information aided parental decision-making and reduced feelings of anger and distress [44].

Parents who received only basic information but with whom the implications of their decisions were discussed, whose questions were answered, and whose emotions were taken into account also felt empowered to make decisions and were satisfied with the service delivery, as their interactions with medical professionals had resulted in a sense of trust [44]. Parents felt that they were able to make informed decisions when they were sufficiently informed of their child’s condition and prognosis [54]. Receiving information from medical staff was cited as being important by parents who also appreciated the respect shown for their decisions [48]. It was also important to parents for information about the bleak prospects facing the child to be balanced with expressions of hope for a better outcome [55,56]. The stance healthcare professionals adopted towards medicine and technology had an impact on parents’ perceptions and decisions; for example, parents were more likely to accept the limitations of existing treatments when healthcare professionals displayed humility and acknowledged themselves that treatments and technology cannot always bring about the desired results [50]. Finally, decision-making was facilitated when parents were not pressured and when they were given ample time [53,55].

### What features do parents find unhelpful/damaging in the decision-making process?

Parents were often confronted with their inability to obtain information about their child, as they did not know what questions to ask [24,27,57], particularly in the initial stages [58].

“I don’t know if you would even know what sort of information you need”, [24].

In other cases, parents actively sought not to become familiar with potential future impairments and preferred to deal with situations as and if they arose [29]. Parents also indicated that receiving (oftentimes conflicting) information from numerous healthcare professionals was confusing and concerning [40,43-45,55,59,60]. One study found that, amongst other factors, straightforward explanations about the child’s condition and tangible proof of a bad prognosis assisted in parental decisions relating to withdrawal of treatment [61]. In relation to written information provided to parents as part of antenatal counselling, an international study found that written information was not considered to be satisfactory by the majority of respondents [30], but further details relating to the specific aspects that were unsatisfactory were not provided.

Finally, parents attempting to make decisions based on quality of life considerations encountered difficulties using such a criterion [29], while others were able to articulate what, in their minds, constituted quality of life and applied the concept to decisions made in instances where they had time to reflect [32].

Studies have shown that some parents’ judgment of the decision they made changed over time. As time passed, some parents began to wonder whether they had made the wrong decision as a result of their lack of scientific knowledge amongst other things [61].

### What resources have parents used?

When confronted with the news of the health status of their unborn or newborn baby, parents appear to take matters into their own hands in search of reliable information. In one study, published in 2001, parents reported spending up to 20 hours in the first week seeking information by asking questions or referring to books and other materials [58]. The same study found that 90% of parents relied on information from doctors and nurses, 13% obtained information from classes, and 13% from videos [58]. In addition to relying on healthcare staff to provide information, parents also relied on them to recommend appropriate materials such brochures, books, and community support groups [57].

The internet was a source of information for many parents in recent studies [34,55,62-68]. The majority of parents appeared to rely on healthcare professionals for information but simultaneously consulted websites for information [67,68], even if they recognized that information on the internet was not reliable [68]. A 2006 study found that only 8% of the study participants (n=101) relied heavily on the internet and books for information [66].

“After we were given that information, that was it. There was no more information given, there was no one else to talk to. And that was it. We went to the Internet, we went to the library, we researched everything about radiation, stem cell transplant, everything we could find” [62].

Other sources that parents accessed included magazines and television shows [34,55], books [49,57,58,62,66], research [29], and even unconventional sources such as mediums and dreams [29].

### What suggestions are put forward by parents?

There was overlap between what parents identified as impeding their ability to make end-of-life decisions or their unmet needs and the suggestions they put forward. The majority of suggestions made by parents in the reviewed literature related to information sharing and various aspects of communication. Presented below are the various facets that relate to the manner with which information is communicated as well as the information itself.

#### Honesty

Parents wanted to be advised of the uncertainty doctors face [25,40,52,69].

“Give parents more of a context for the experience-let them know, while you don’t know the exact outcome for any particular child, more often than not this is the course of the next few hours, days, weeks, months … this would give us a better perspective to face and make better decisions down the road rather than responding to the limited situation/crisis immediately in front of us” [40].

Some parents identified the importance of receiving honest information as being linked to both their ability to make informed decisions but also their ability to cope better following their child’s death [40,61]. In another study, honest information was identified as one of the eight priorities that primary caregivers perceive as having [70]. Honest responses to mothers’ queries were also seen as a key communication priority by mothers with babies in a neonatal intensive care setting [71].

#### Hope and false hopes

Even though parents wanted information delivered truthfully, there was a strong preference for all information to be delivered with an element of hope [36,46,53,69]. Importantly, parents were able to distinguish between hope and false hopes [25,41,45,55,56,69], the latter of which was defined as “…information that was too optimistic and given simply to make the parent feel better at the time without also acknowledging or preparing the parent(s) for any potential negative outcomes” [55].

“Doctors need to relay medical facts honestly but always allow for a glimmer of hope, even if only for a miracle. The doctors who best connected with S always had hope…” [46].

#### Sensitivity

When difficult information was delivered, parents wanted this to be done in a compassionate and sensitive manner [23,42,46,63,72].

“…Be sensitive, honest, cautious about word choice” [46].

#### Time, timing, and timeliness of information

Some parents felt that information was scarce immediately after the birth or transfer of their child and expressed the need for more information at that stage [23]. They also felt that discussions about their child should be timely [25,71], and that there should be a number of meetings between parents and medical experts when end-of-life decisions need to be reached [73], as well as frequent updates on their child’s progress [59,71].

Parents in one study felt that discussions about disability should have taken place much earlier in the process, including antenatally, and indicated that such discussions would have better prepared them, but not changed their treatment decisions [29]. In another study, parents, interviewed six months after discharge from intensive care, reflected that they would have benefitted from more information about how their child would impact on their lives and urged parents to ask more questions rather than allow medical professionals to have complete control [27].

Time was also relevant in relation to the comprehension and acceptance of medical information with parents expressing the view that they needed time to process information [43]. It was felt that the most appropriate time to deliver information to parents was during the doctor’s rounds but a number of parents felt that separate times outside the rounds would also be appropriate for them to be given information [66]. When discussing issues with parents, it was felt that medical professionals needed to spend more time with them [30], in order to discuss issues in a calmer, less rushed manner as well as listen to what parents had to say [40]. Some parents felt that information should only be provided when the parent is ready to receive it given its gravity [74].

#### Involvement in decisions

Some parents believed that decisions should not be made without the involvement of medical professionals [60]. Parents warned medical practitioners against thinking that the provision of information on a single occasion was enough for parents to come to a decision about end-of-life care [72]. End-of-life decisions were described as an ‘evolving process’ which requires the reiteration of information to allow parents to absorb and process it [72]. Parents needed to ask questions and seek clarifications [27,72,75-77], which would enable their thinking to evolve and for them to come to a decision for their child [72]. When engaging parents in the decision-making process, parents thought it was important for staff to ascertain the level of involvement parents wish to have in this process [74].

#### Medical jargon

In numerous studies parents requested simplified language. They suggested that the likely outcomes for their child should be explained in non-technical language [37,77], as well as the nursing care provided to their child [71].

“When doctors would explain, the words kept getting bigger and bigger; it would be helpful to have someone to break it down into more simple explanations.” [37]

An international study found differences in parental preferences regarding the use of jargon [30]; while the vast majority of parents in Kuala Lumpur, Singapore, and Hong Kong felt that the use of simpler language was important, parents from San Francisco, Melbourne, and Tokyo (all below 50% of parents included in the study) were slightly less inclined to think that simplified terminology would lead to better communication [30]. Parents in another study indicated that in addition to a sensitive manner of delivery, it was important for information to be delivered in simple terms [46]. One mother went as far as suggesting that blunt terms such as *die, retarded, crippled* be used, as the terms most often used (e.g. expire, developmentally delayed, brain damage) could not be easily processed when in a state of shock [53].

#### Complete information

A desire for complete information was expressed by a number of parents in various studies [24,27,41,70]. Some parents referred to the need for additional information relating to autopsies and the circumstances under which death occurred [53,59]. Parents also required more detailed information on the outcomes envisaged for their child [30,70,76,77], and appreciated information regarding the options available to them [69].

#### Written materials

Amongst the suggestions parents offered for improved communication and understanding of the information exchanged in relation to end-of-life decision-making was that written information be provided to enhance retention [56], and that information presented in pamphlets or booklets be made available [37]. Fathers in another study suggested that written information about common medical conditions and online resources should be provided [51].

“Maybe if I had like some literature to read, just a little pamphlet to say, okay I read up on it and I say okay I understand a little bit more now, you know. Maybe I feel a little bit more strongly this way than that way” [56].

## Discussion

This meta-synthesis has highlighted the information needs of parents facing end-of-life decisions and aimed to assist in determining what their written information needs are. These information needs are varied and complex, as end-of-life decisions engage the full range of a human being’s most fundamental aspects of existence by involving cognitive, emotional, ethical, and spiritual/religious dimensions. The complexity of such decisions impacts on parents and healthcare professionals alike.

The most frequent complaint, request, and suggestion revealed in this meta-synthesis of findings related to the need for more information, particularly, information specific to their child. Another common request related to the manner with which difficult information is delivered, which was not always seen to be sensitive. Training of healthcare professionals in communication skills may help them to provide information sensitively and empathically [78] but written resources sensitively written can also provide a model for the kind of language and the tone that is suitable for parents experiencing the greatest distress they will ever know. Also assisting with communication might be support people, such as social workers, interpreters, family members, chaplains etc., as they may assist parents to express their questions and concerns. The availability of reliable written resources can be invaluable to those supporting parents in their efforts to make the best decisions for their child.

It is important to take into account parents’ experience with the provision of information from healthcare professionals. Parents reported that, as a result of the extreme emotional strain and the wealth of medical details they were suddenly exposed to, they encountered difficulties processing and making sense of information provided by healthcare professionals [23-28]. In fact, parents often shift clinical conversations to less distressing topics, as they are emotionally unable to bear discussing end-of-life decisions for extended periods [79]. Clinicians sometimes misconstrue these diversions as a lack of appreciation of the gravity of the situation and attempt to revisit the clinical discussion thus adding to parents’ discomfort [79]. The fact that parents privately contemplate the likelihood that their child may die [79] lends support to the development of written materials to aid end-of-life considerations.

In addition to information provided by healthcare professionals, parents often seek information from a variety of secondary sources, including (but not limited to) scientific literature [79]. Written resources have been shown to improve understanding and reduce long-term distress in families of critically ill adult patients [11,12]. There are therefore several potential advantages to written materials for end-of-life discussions in contexts relating to children. Such resources can be re-visited as frequently as parents require so that they are better able to absorb the information and can begin to build a deeper understanding of the medical terminology, and the medical facts and their implications, especially in the initial stages of their exposure to intensive care and end-of-life decisions. Written resources also have the benefit of being available at the moment when parents need to access them whether this is in the hospital setting or in the privacy of parents’ own homes. Furthermore, they play an important role in reinforcing information provided by healthcare professionals [71] and can be used as discussion initiators with healthcare professionals as well as other members of the family. Written resources cannot replace the invaluable contribution made by healthcare professionals but they may be of assistance to parents when the availability of healthcare professionals is temporarily limited. In line with findings from this meta-synthesis, written materials need to be clearly and sensitively written with medical jargon explained in simple language.

The literature reviewed indicated some of the areas that may be useful for written (or other) material to cover. A written resource cannot provide child-specific information but can provide clear, simple-to-understand general medical information. This may assist both in a better understanding of the intricacies of intensive care treatments and may simultaneously empower parents to ask for more information from healthcare professionals. A common obstacle cited in the literature is that parents simply lack the means to ask questions whose answers may impact on their decision-making [24,27,57,58]. In addition to the factual content, written resources with suggested questions which commonly arise in end-of-life discussions may provide a means for parents to articulate their queries and concerns thus improving communication between healthcare professionals and parents.

There are a number of issues that cause significant distress to parents but may not always be openly discussed with healthcare professionals. Examples include the uncertainty that often prevails in prognoses and conflicting medical opinions about treatment options, which causes parents great distress and concern [25]. Such issues might be almost impossible for healthcare professionals and parents alike to broach in conversation. Consideration and an explanation of the complexities of some prognoses and treatment decisions in written materials could potentially address some of the unspoken queries and provide an explanation regarding the limitations of medical treatments and conflicting views that sometimes arise in medicine.

Parents have indicated clearly in the studies considered in this meta-synthesis that religious, cultural beliefs, personal values, and certain stances, such as preserving hope, are very important in their decision making [32,36-39,44]. Despite their importance, such topics can be difficult to discuss openly. Written resources intended for parents could probably not enter into lengthy considerations of such issues. They could, however, acknowledge their importance, normalize their presence, provide helpful clarifications, and encourage discussion with healthcare professionals.

Written resources can also assist in bringing others’ experiences to the reader. Numerous parents expressed the view that communicating with parents who have had the same or similar experiences would be beneficial [47,48]. Written materials can cater to this need by providing authentic parent voices speaking of their experience and the issues they faced. The knowledge that other parents have experienced the same or similar problems and circumstances must bring some comfort.

While this meta-synthesis focused on parental needs, it is possible that written resources about end-of-life decision-making for parents of seriously ill or dying children might also be useful to experienced and trainee healthcare professionals both in relation to the content of such resources but also in relation to the tone and language adopted.

### Limitations of the meta-synthesis

Only one of the authors conducted the searches. The quality of the primary studies included in the meta-synthesis was not appraised for the reasons provided. The literature search includes papers up to 2012 and is reflective of the period during which work was undertaken to compile written resources for parents. Furthermore, the challenge of extrapolating the requirements for written materials from feedback regarding general information needs must be acknowledged.

## Conclusion

The meta-synthesis of predominantly qualitative literature helped to identify gaps in the provision of information, and the information needs of parents who face end-of-life decisions. The points discussed are of general relevance for communication, though we have focused specifically on the question of written information. The meta-synthesis has utility for clinical practice. This work can also be used to guide the development of written materials to support parents, such as the those which our collaborative research group have recently developed [18,19], (See Additional file 2 for topics covered in online resource). However, further research is needed to evaluate whether such written resources meet the needs of parents, assist them in their decision-making, and have a positive impact on their long-term wellbeing.

## Additional files

Additional file 1: Table S1. Studies included in meta-synthesis. Provides information about all the studies included in the meta-synthesis of findings relating to communication or information-related features that parents report as being helpful or unhelpful during end-of-life decision-making.
Additional file 2: **Topics covered in the parental resource*****Caring Decisions.*** Provides an overview of the topics addressed in the comprehensive online resource developed for parents (and clinicians) involved in making end-of-life decisions for the seriously ill or dying child.
