# Supplementary material for: What information do parents need when facing end-of-life decisions for their child? A meta-synthesis of parental feedback
Source: BMC Palliat Care. 2015 Apr 30;14:19. doi: 10.1186/s12904-015-0024-0 (PMC4424961; doi:10.1186/s12904-015-0024-0)
Supplement: Additional file 1: Table S1. — Studies included in meta-synthesis. Provides information about all the studies included in the meta-synthesis of findings relating to communication or information-related features that parents report as being helpful or unhelpful during end-of-life decision-making. [file 12904_2015_24_MOESM1_ESM.pdf]

Table S1. Studies included in meta-synthesis

	Study	Design	Key focus	Participants	Country	Patient group
1	Branchett, K. and J. Stretton (2012). "Neonatal palliative and end of life care: What parents want from professionals."	Inductive study utilizing an internet parental support forum. Thematic analysis.	Parents' experiences in neonatal palliative care and lessons to be learnt	54 mothers and 3 fathers who had lost a child in the neonatal period	UK	neonates
2	Hellmann, J., C. Williams, et al. (2012). "Withdrawal of artificial nutrition and hydration in the Neonatal Intensive Care Unit: Parental perspectives."	Retrospective chart review and parental survey including open-ended comments. Analysed using simple statistics and theme generation for qualitative data.	Parental perspectives on withdrawal of artificial nutrition and hydration in NICU	11 sets of parents whose babies died after withdrawal of hydration & nutrition	Canada	neonates
3	Pepper, D., G. Rempel, et al. (2012). "More Than Information: A Qualitative Study of Parents' Perspectives on Neonatal Intensive Care at the Extremes of Prematurity."	Interpretive descriptive study, semi-structured interviews. Major themes identified, commonalities explored, and interrelationship among themes investigated and described.	Parental perceptions of decision making concerning their extremely premature newborns	7 parents of preterm infants who were born at 24-26 weeks' gestation	Canada	neonates
4	Roscigno, C. I., T. A. Savage, et al. (2012). "Divergent views of hope influencing communications between parents and hospital providers." Qualitative Health Research 22(9): 1232-1246.	Semi-structured interviews before and after delivery. 4 interview types (prenatal, postnatal, stillbirth, and end of life). Code-based descriptive case summary used for construction of matrices for each type of interview, allowing for comparison of data within and across cases.	Evaluation of the varying notions of hope between parents who were at risk of a peri-viable delivery and their healthcare providers	40 mothers with possible preterm delivery before 26 weeks, 14 of their partners, and 71 doctors, neonatal nurses, and staff nurses.	USA	neonates
5	Shaw, A. (2012). "'They Say Islam Has A Solution For Everything, So Why Are There No Guidelines For This?' Ethical Dilemmas Associated With The Births And Deaths Of Infants With Fatal Abnormalities From A Small Sample Of Pakistani Muslim Couples In Britain."	3 case studies taken from qualitative research involving 66 families	Case studies of Muslim parents' ethical dilemmas concerning the termination of pregnancy, the management of childbirth, and the withdrawal of life-support from infants in special care.	3 couples	UK	fetus /neonate
6	Tan, J. S., S. L. Docherty, et al. (2012). "Addressing parental bereavement support needs at the end of life for infants with complex chronic conditions."	Longitudinal, qualitative, descriptive design. Extreme case sampling used to select 7 cases. Content analysis.	The bereavement experience of parents whose infants die in acute care settings with a complex chronic condition	14 parents of 8 infants	USA	infants
7	Black, B. P. (2011). "Truth telling and severe fetal diagnosis: A virtue ethics perspective."	Longitudinal ethnography examining parents' experiences across 3 available care options: termination, routine obstetric care, and perinatal end-of-life care. Content analysis. Data matrices constructed for data comparison.	How parents create meaning and truth in the context of the prenatal diagnosis of severe impairment	15 women and 10 male partners with a severe fetal diagnosis (3 lots of interviews conducted with each)	USA	fetus/ neonates

	Study	Design	Key focus	Participants	Country	Patient group
8	Caeymaex, L., M. Speranza, et al. (2011). "Living with a crucial decision: A qualitative study of parental narratives three years after the loss of their newborn in the NICU."	Retrospective longitudinal design. Face-to-face & telephone interviews. Discourse analysis.	Parents' experience of the end-of-life decision-making process	53 face-to-face interviews and 80 telephone interviews conducted with 164 individuals 3 years after the death of their child	France	neonates
9	Côté-Arsenault, D. and E. Denney-Koelsch (2011). ""My baby is a person": Parents' experiences with life-threatening fetal diagnosis."	Qualitative descriptive study using open ended questions. Thematic analysis.	Parents' experience of pregnancy with a life-threatening diagnosis to understand their needs for perinatal palliative care services, and establish the feasibility and acceptability of conducting intensive interviews of pregnant women and their partners during their pregnancy	2 women and 3 couples during pregnancy or just after birth	USA	fetus/ neonates
10	Hexem, K. R., C. J. Mollen, et al. (2011). "How parents of children receiving pediatric palliative care use religion, spirituality, or life philosophy in tough times."	Prospective cohort study on parental decision-making for children receiving pediatric palliative care-interviews. Grounded theory.	Enquiry into how religion, spirituality, or life philosophy (RSLP) was helpful to parents in difficult times (including decision-making)	73 parents	USA	children (aged 1-24)
11	Knapp, C., V. Madden, et al. (2011). "Information seeking behaviors of parents whose children have life-threatening illnesses."	Cross-sectional, telephone survey. Statistical analyses.	Associations between parental characteristics and preferred health information sources & parents' e-health literacy	129 parents with children in a pediatric palliative care program	USA	children
12	Michelson, K. N., L. Emanuel, et al. (2011). "Pediatric intensive care unit family conferences: One mode of communication for discussing end-of-life care decisions."	Retrospective qualitative study. In-depth, semi-structured focus groups and interviews. Thematic analysis.	Pediatric palliative care conferences & family conferences (FC) for discussions about end-of-life decision-making.	18 parents of children who died in the pediatric intensive care unit and 48 pediatric intensive care unit professionals	USA	children
13	Moro, T. T., K. Kavanaugh, et al. (2011). "Parent decision making for life support for extremely premature infants: From the prenatal through end-of-life period."	Five cases with prenatal, postnatal and end-of-life interviews, and medical record data extraction. Data were coded based on the Ottawa Decision Support Framework. Summaries and matrices were generated for prenatal, postnatal and end-of life interviews. Comparison of data within and across cases and groups, i.e. parents, nurses, and physicians, were conducted.	How parents make life support decisions from the prenatal period through death.	5 cases comprising 5 mothers, 4 neonatologists, 3 nurses, and 1 neonatal nurse practitioner, drawn from a larger collective case study	USA	neonates
14	Thiele, P. (2011). "Going against the grain: Liam's story."	Case study, self-reported by mother	The experience of one mother losing her baby	1 mother	Aus	neonates

	Study	Design	Key focus	Participants	Country	Patient group
15	Davies, B., N. Contro, et al. (2010). "Culturally-sensitive information-sharing in pediatric palliative care."	Retrospective design of grounded theory analysis. Questionnaire with 21 questions, (summated Likert-type scale and descriptive response)	Chinese and Mexican American parents' perspectives on communication with health care providers in palliative care	36 parents (26 Mexican American and 10 Chinese American) from 28 families who experienced death of child (<20 years)	USA	children<20 years in pediatric palliative care
16	Einaudi, M. A., Le Coz P. et al. (2010). "Parental experience following perinatal death: Exploring the issues to make progress."	Qualitative descriptive survey - questionnaire based on the Delphi method, semi-directed interviews and written survey. Secondary analysis of ethical principles encountered by participants.	To understand parental attitudes, needs and ethical issues associated with perinatal death, to assist in the development of interventions for bereaved families	10 mothers and 2 fathers (3 interviews with mothers, rest mail responses)	France	neonates
17	Kavanaugh, K., T. T. Moro, et al. (2010). "How nurses assist parents regarding life support decisions for extremely premature infants."	Qualitative, longitudinal, collective case study – semi-structured interviews pre- and postnatally and extraction of medical chart data.	Nurse behaviours that assist parents make decisions about very premature babies	40 cases (40 mothers, 14 fathers, 42 physicians, 17 obstetric nurses, 6 neonatal nurses, and 6 neonatal nurse practitioners).	USA	neonates
18	Einarsdóttir, J. (2009). "Emotional experts: Parents' views on end-of-life decisions for preterm infants in Iceland.	Qualitative research using open ended and semi-structured interviews.	Parents' perceptions about whether and when treatment should be withdrawn and who should decide	28 mothers and 25 fathers	Iceland	neonates
19	Michelson, K. N., T. Koogler, et al. (2009). "Parental views on withdrawing life-sustaining therapies in critically ill children."	Quantitative and qualitative analysis of semi-structured one-on-one interviews.	Parental willingness to consider withdrawing life-sustaining therapies and the influences of quality of life, physician estimated prognosis, patient suffering, and financial burden on such decisions.	70 parents	USA	children
20	Williams, C., J. Cairnie, et al. (2009). "Construction of a parent-derived questionnaire to measure end-of-life care after withdrawal of life-sustaining treatment in the neonatal intensive care unit."	Semi-structured interviews used to develop a survey instrument for social workers.	Parents whose infants had undergone withdrawal of life-sustaining treatment in the NICU were interviewed to obtain their views on helpful practices to build an instrument to measure important aspects of Withdrawal of Life Support Treatment practices.	11 parents whose infants had undergone withdrawal of life-sustaining treatment in the NICU 48 social workers completed questionnaire	Canada	neonates
21	Arockiasamy V, Holsti L, and Albersheim S, (2008) "Fathers' experiences in the neonatal intensive care unit: A search for control."	Semi-structured interviews with open-ended questions. Coded using the constant comparative method of content analysis.	Level of comfort with or concerns about staff communication regarding their infant, about accessing information, and about more general perceptions of their experience in the NICU.	16 fathers of very ill and/or very preterm infants who had been in the NICU for 30 days	Canada	neonates
22	Boss, R. D., N. Hutton, et al. (2008). "Values parents apply to decision-making regarding delivery room resuscitation for high-risk newborns."	Qualitative multi-center study. Parents interviewed about their prenatal decision-making. Maternal medical charts reviewed for documented discussions about delivery room resuscitation. Content analysis.	Values guiding parental decision-making regarding delivery room resuscitation for infants born extremely prematurely or with potentially lethal congenital anomalies.	26 mothers	USA	neonates

	Study	Design	Key focus	Participants	Country	Patient group
23	Meert, K. L., S. Eggly, et al. (2008). "Parents' perspectives on physician-parent communication near the time of a child's death in the pediatric intensive care unit."	Secondary analysis of a qualitative interview study. Detailed notes of parent interviews compared and used for identification of themes.	Positive and negative ways of communicating bad information to parents regarding their child's terminal illness and death	56 parents of 48 children who died in the PICU 3-12 months prior to study.	USA	children
24	Widger, K. and C. Picot (2008). "Parents' perceptions of the quality of pediatric and perinatal end-of-life care."	Face-to-face or telephone interviews with specially developed survey. Frequencies of responses to questions were determined and problem areas identified. Responses to open-ended questions summarized.	Quality of care provided (including communication of information) around the death of the child (12-24 months prior to study commencement)	38 families (primarily mothers)	Canada	infants, children, adolescents
25	Armentrout, D. C. (2007). "Holding a Place: Parents' Lives Following Removal of Infant Life Support."	Grounded theory study. Face-to-face and telephone interviews.	Parental decision-making to discontinue life support and identification of the process that allows them to then move forward with their lives after the infant's death.	15 parents (4 couples and 7 mothers) drawn from support group for parents who experienced neonatal death	USA	neonates
26	Brosig, C. L., R. L. Pierucci, et al. (2007). "Infant end-of-life care: The parents' perspective."	Revised Grief Experience Inventory (RGEI) completed by parents and a semi-structured interviews regarding their infants' end-of-life care. Interviews rated using the Post-Death Adaptation Scale (PDAS).	Factors important to parents in their infant's end-of-life care.	19 families whose infant (less than 1 year old) had died	USA	neonates/ infants
27	Carnevale, F. A., P. Canoui, et al. (2007). "Parental involvement in treatment decisions regarding their critically ill child: A comparative study of France and Quebec."	Grounded theory qualitative methodology. Semi-structured interviews were conducted. (Comparison between French and Quebec cohorts)	To determine who assumes decision-making responsibility for treatment decisions and how this relates to subsequent parental experience.	31 parents of critically ill children; 9 physicians & 13 nurses who cared for their children	France & Canada	children
28	Konrad, S. C. (2007). "What parents of seriously ill children value: Parent-to-parent connection and mentorship."	Psychological phenomenological design with open-ended interviews. Ongoing and comparative method of thematic analysis.	Parent-to-parent mentorship for parents of seriously ill or dying children	12 mothers of seriously ill children, including those whose children had died	USA	children (birth-age 15)
29	Rini, A. and L. Loriz (2007). "Anticipatory Mourning in Parents With a Child Who Dies While Hospitalized."	An exploratory design used to answer questions in focused semi-structured interviews. Thematic analysis(?)	Parents' descriptions of their experiences surrounding the death of their child and the role of anticipatory mourning	11 parents whose child died during hospitalisation	USA	children (birth-age 12)
30	Alderson, P., J. Hawthorne, et al. (2006). "Parents' experiences of sharing neonatal information and decisions: Consent, cost and risk."	Ethnographic research. Semi-structured taped interviews. Thematic analysis (?)	Parents' experiences of sharing information and decisions with neonatal staff	parents of 80 babies with definite or potential neuro-developmental problems and 40 senior practitioners	UK	neonates
31	Berg, S. (2006). "In their own voices: families discuss end-of-life decision making--part 2."	Personal narrative	A mother's interactions with healthcare providers when faced with difficult decision-making about her toddler.	1 mother	USA	toddler

	Study	Design	Key focus	Participants	Country	Patient group
32	Dokken, D. L. (2006). "In their own voices: families discuss end-of-life decision making--part 1. Interview by Elizabeth Ahmann."	Personal narrative	The story of a mother's interactions with health care providers when faced with end-of-life decision making for her baby	1 mother	USA	neonate
33	Kowalski, W. J., K. H. Leef, et al. (2006) Communicating with parents of premature infants: who is the informant?	A 19-item questionnaire comprising multiple choice questions, yes/no questions, 5-point Likert scale questions, and open-ended questions. Statistical analysis.	Examines sources of information, who provides it, and parents' expectations regarding obtaining information in NICU	101 parents of infants 32 weeks or younger prior to discharge from the NICU	USA	neonates
34	Meyer, E. C., M. D. Ritholz, et al. (2006). "Improving the quality of end-of-life care in the pediatric intensive care unit: Parents' priorities and recommendations."	Qualitative study based on parental responses to open-ended questions on anonymous, self-administered questionnaires. Content analysis.	Identification and description of priorities and recommendations for end-of-life care and communication from the parents' perspective.	56 parents	USA	neonates-18 years of age who died
35	Payot, A., S. Gendron, et al. (2006). "Deciding to resuscitate extremely premature babies: How do parents and neonatologists engage in the decision?"	Interpretive qualitative method (informed by Guba and Lincoln's hermeneutic and dialectical approach). In-depth semi-directed interviews immediately following neonatal consultation.	How parents and neonatologists engage in decision-making in a context of imminent and unplanned delivery at the threshold of viability	8 couples<br>& 4 neonatologists	Canada	fetus<br>/neonates
36	Kavanaugh, K., T. Savage, et al. (2005). "Life support decisions for extremely premature infants: Report of a pilot study."	Qualitative collective case study –pilot (one prenatal, one postnatal, and one post death). Ottawa framework used for data analysis. Data were compared within and across each case.	Decision making and the decision support needs of parents, physicians, and nurses regarding life support decisions made prenatally and postnatally for very premature infants	6 cases (6 mothers, 2 fathers, 6 physicians, and 2 nurses)	USA	neonates (premature)
37	Keenan, H. T., M. W. Doron, et al. (2005). "Comparison of Mothers' and Counselors' Perceptions of Predelivery Counseling for Extremely Premature Infants."	Descriptive study. Mothers interviewed by telephone using a standardized interview format developed for this study and their counsellors. Thematic analysis (?)	Mothers' and Counselors' perceptions of their roles in decision-making regarding resuscitation and mothers' and counselors' satisfaction with the counseling and decision-making process.	33 mothers and their counselors	USA	neonates (premature)
38	Partridge, J. C., A. M. Martinez, et al. (2005). "International comparison of care for very low birth weight infants: Parents' perceptions of counseling and decision-making."	Quantitative study using interview tool developed for this study. Half-hour structured interviews. Derived descriptive statistics (frequencies) and subgroup comparisons from survey responses.	Comparison of parental perceptions and satisfaction with antenatal counseling and delivery room resuscitation (factors taken into consideration in decisions, and acceptance of parental decision-making) in 6 Pacific Rim countries and 2 Californian hospitals. Sample comprised mostly parents whose infants survived.	Parents interviewed at each site: Taiwan 45, Hong Kong 42, Singapore 56, Kuala Lumpur 37, Tokyo 31, Melbourne 51, San Francisco 65	Australia, Hong Kong, Japan, Malaysia, Taiwan, Singapore2 sites in San Francisco	neonates (premature)
39	Sharman, M., K. L. Meert, et al. (2005). "What influences parents' decisions to limit or withdraw life support?"	Prospective, qualitative pilot study. In-depth, semi-structured interviews. Qualitative data examined to identify factors important to parents during their decision-making process. Coding dictionary developed.	Factors influencing parents in their decision to forego life support for their children.	14 parents of ten children whose pediatric intensive care unit physician had made a recommendation to limit or withdraw life support.	USA	children

	Study	Design	Key focus	Participants	Country	Patient group
40	Orfali, K. and E. J. Gordon (2004). "Autonomy gone awry: A cross-cultural study of parents' experiences in neonatal intensive care units."	Comparative ethnographic approach. In-depth, semi-structured interviews.	Parental decision-making to discontinue life support in France (Units A & C) and US (Unit B)	75 mothers of critically ill or deceased babies	France & USA	neonates
41	Pector, E. A. (2004). "Views of bereaved multiple-birth parents on life support decisions, the dying process, and discussions surrounding death."	Grounded theory approach used for qualitative data - a narrative e-mail survey assessing many facets of bereavement	Experiences of bereaved parents of multiples with resuscitation and life-support discussions, the death process, and conversations with health-care professionals about death.	71 bereaved parents of multiples recruited from Internet support groups (4 of whom were fathers)	North America	fetuses/ neonates
42	Dhillon A S, Albersheim S G, Alsaad S, Pargass N S, and Zupancic J A F, (2003) "Internet use and perceptions of information reliability by parents in a neonatal intensive care unit."	Interviews utilising standardised questionnaire. Statistical analyses including logistic regression analyses	To assess internet use and perceptions of information reliability by parents in a neonatal intensive care unit.	90 parents of the babies admitted to the NICU	Canada	neonates
43	Lundqvist, A., T. Nilstun, et al. (2003). "Neonatal end-of-life care in Sweden: the views of Muslim women."	Interviews using a standardized questionnaire with open-ended questions about care before birth, directly after birth, and during and after the death of the infant. Content analysis was performed on the data.	Muslim women's views on neonatal end-of-life in Sweden	11 women (including 1 couple) (women from Iran, Iraq, Somalia, Turkey) experience with fetal impairment or neonatal death not necessary	Sweden	hypothetical scenario involving neonates
44	Bialoskurski M M, Cox C L, and Wiggins R D, "The relationship between maternal needs and priorities in a neonatal intensive care environment."	The theoretical framework for the study was based on symbolic interactionism Quantitative survey. Analysed with multivariate analysis	To investigate the nature and organization of maternal needs and priorities in a neonatal unit.	209 mothers with premature infants.	UK	neonates
45	Brinchmann, S. B., R. Førde, et al. (2002). "What Matters to the Parents? a qualitative study of parents' experiences with life-and-death decisions concerning their premature infants."	Descriptive study design using in-depth interviews. The comparative method (grounded theory) was used to analyse the data.	Parents' participation in life-and-death decisions concerning their very premature and/or critically ill infants in hospital neonatal units	35 parents of 26 children (Ten of the infants died; 16 were alive at the time of the interview.)	Norway	neonates
46	Contro, N., J. Larson, et al. (2002). "Family perspectives on the quality of pediatric palliative care."	Qualitative needs assessment undertaken to inform the development of a Pediatric Palliative Care Program	Experiences and their suggestions for improving the quality of end-of-life care were elicited from parents with involvement in end-of-life care	68 family members of 44 deceased children (English and Spanish speaking)	USA	children
47	Davies, B. and S. Connaughty (2002). "Pediatric End-of-life Care: Lessons Learned From Parents."	The parental questionnaire consisted of 21 questions, most requiring both a response to a summated Likert-type scale and a descriptive response. Design not described.	Parental insights into the meaning of optimal care for dying children	45 parents of children who died during a 2-year period	USA (and possibly Canada-unspecified)	children

	Study	Design	Key focus	Participants	Country	Patient group
48	Meyer, E. C., J. P. Burns, et al. (2002). "Parental perspectives on end-of-life care in the pediatric intensive care unit."	Self-administered questionnaire with 28 Likert format questions and five open-ended questions. Statistical analysis. Analysis of the open-ended, qualitative questions not done for this paper but some quotes in Discussion.	Parental perspectives on priorities for quality end-of-life care	56 mother and fathers (36 mothers, 20 fathers) of children who had died after withdrawal of treatment	USA	children
49	Brazy, J. E., B. M. Anderson, et al. (2001). "How parents of premature infants gather information and obtain support."	Descriptive cross-sectional study (in-depth structured interviews followed by detailed questionnaire designed to validate initial impressions and quantify responses). Data grouped into categories based on questions.	Process by which parents of premature infants seek information, the kinds of information they seek, and the resources they use to meet their educational and support needs	19 parents of premature infants interviewed and questionnaires with 64 parents who had previously given birth to premature babies who were discharged alive	USA	neonates
50	McHaffie, H. E., A. J. Lyon, et al. (2001). "Deciding on treatment limitation for neonates: The parents' perspective."	In-depth face-to-face semi-structured interviews. Method of analysis not described in detail.	Parents' perceptions of withdrawal/withholding treatment	Interviews with 108 parents at 3 months after the death of the baby and then 90 parents at 13 months after the death of the baby	Scotland	neonates
51	Wocial, L. D. (2000). "Life support decisions involving imperiled infants."	Descriptive, qualitative study with open-ended telephone and face-to-face interviews. Content analysis.	Parents' perception of their experiences in the neonatal intensive care unit (NICU) when faced with the dilemma of withholding and/or withdrawing treatment from their infants.	20 parents whose babies received treatment in a neonatal intensive care unit and died	USA	neonates
52	Kavanaugh, K. (1997). "Parents' experience surrounding the death of a newborn whose birth is at the margin of viability."	Descriptive, using an eidetic phenomenologic approach. Face-to-face and telephone interviews 4 and 15 weeks after the loss of the baby.	Parental experience of their baby's death	8 parents (5 mothers and 3 of their husbands)	USA	neonates
53	Scott, L. D. (1998). "Perceived needs of parents of critically ill children."	Descriptive, comparative. Structured interview technique.	Parental needs identified via the Critical Care Family Needs Inventory (a 45-item instrument developed to quantify the significance of perceived family needs- modified for pediatrics)	21 carers (19 mothers, 1 grandmother, 1 aunt) and pediatric critical care nurses (n = 17).	USA	children/ adolescents
54	Kirschbaum, M. S. (1996). "Life support decisions for children: What do parents value?"	Phenomenological approach. An open-ended, non-schedule standardized interview format. Phenomenological analytic approach.	Issues that factored into parents' decision-making in relation to withdrawing or withholding life sustaining treatment	20 families (two interviews conducted with both parents) families who had faced withdrawal/ withholding of treatment decision in the previous 6 to 12 months	USA	children
55	Pinch WJ, Winifred J, Spielman ML. (1996) “Ethics in the Neonatal Intensive Care Unit: Parental Perceptions at Four Years Postdischarge.”	Multistage, exploratory descriptive study using a qualitative approach. In-depth interviews and the use of content analysis.	Families’ perceptions of moral conflict and ethical decision making related to their NICU experience	23 families forming phase 3 of a longitudinal study	USA	neonates

	Study	Design	Key focus	Participants	Country	Patient group
56	Pinch WJ, Spielman ML. (1993) “Parental perceptions of ethical issues post-NICU discharge. Western journal of nursing research.”	Multistage, exploratory descriptive study using a qualitative approach. In-depth interviews and the use of content analysis.	Parental perceptions of ethical issues , including treatment decision making , 6 months after the infants' discharge from NICU	28 families (28 mothers, 19 fathers, 1 grandmother care giver)	USA	neonates
57	Pinch WJ. (1990) “Looking back: Five families share their views of ethical decision making in the NICU.”	Qualitative, phenomenological approach. Interviews using a modification of Lyon’s semi-structured guide. Content analysis.	Five families' descriptions of their experiences with neonates in the intensive care nursery provide a glimpse into their perceptions of ethical problems and moral actions.	Five families (2 husbands also participated)	USA	neonates
58	Pinch, W. J. and M. L. Spielman (1990). "The parents' perspective: ethical decision-making in neonatal intensive care."	Qualitative, phenomenological approach. Semi-structured interviews using a moral conflict interview guide. Content analysis.	Parents' view of ethical decision-making responsibility	32 families with critically ill babies in a neonatal intensive care unit	USA	neonates
