# Supplementary material for: What information do parents need when facing end-of-life decisions for their child? A meta-synthesis of parental feedback
Source: BMC Palliat Care. 2015 Apr 30;14:19. doi: 10.1186/s12904-015-0024-0 (PMC4424961; doi:10.1186/s12904-015-0024-0)
Supplement: Additional file 2: — Topics covered in the parental resourceCaring Decisions. Provides an overview of the topics addressed in the comprehensive online resource developed for parents (and clinicians) involved in making end-of-life decisions for the seriously ill or dying child. [file 12904_2015_24_MOESM2_ESM.pdf]

Topics covered in the parent resource *Caring Decisions*

(available at http://www.rch.org.au/caringdecisions/)

- Introduction
    - How common are decisions about life support for children?
    - Why should I read this handbook?
    - Is this handbook relevant for my child?
    - How should I read this book?
    - Why am I being asked these questions? It is not given to me to decide about life
    - Starting a conversation with your child's doctors
    - 'The bigger picture'
    - There is too much to think about…
    - I don't understand…
    - Support at a stressful time
    - Terms used in this handbook
- How do parents think about end of life decisions
- Life support treatment
    - What is life support treatment?
    - Are there different types of life-support treatment?
- Comfort treatment and palliative care
    - What is comfort treatment and palliative care?
    - Is palliative care just for children who are dying?
    - Can a child have comfort treatment and life support treatment?
- Stopping life support
    - Why would doctors want to stop life support treatments for my child?
    - Are there different reasons for stopping or not starting life support treatment?
    - Is it legal to stop or not start life support treatment?
    - Is stopping treatment euthanasia?
    - What is futile medical treatment?
    - Futile treatment and the dying process
    - Questions you could ask
- Stopping and not-starting
    - Is there a difference between stopping treatment and not starting treatment?
    - What if I still feel uncomfortable stopping life support treatment for my child?
    - What is a 'trial of life support treatment'?
    - Questions that you could ask your child's doctors about treatment:
- Different types of treatment
    - Are decisions different for different life support treatments?
    - Is it OK to stop some life support treatments but continue others?
    - What is a DNAR or AND order?
    - If I agree to a DNAR order for my child will other people know?

    - What will other people think of me if I agree to a DNAR for my child?
    - If I agree to a DNAR will other treatment stop?
    - What if I change my mind?
    - What if I decide that I want doctors and nurses to provide CPR for my child?
    - I want everything to be done
    - Is it OK to stop artificial feeding?
    - Is it legal to stop artificial feeding?
- Doing what is best
    - What is the right thing to do?
    - How do we work out what would be best?
    - Should I ask my child?
    - What if my child doesn't want life support treatment?
- Quality of Life
    - What is 'Quality of Life'?
    - Is it appropriate to use quality of life when making decisions for children?
    - Are all 'quality of life' decisions the same?
    - Are 'quality of life' decisions different in children?
    - What about the 'sanctity of life' approach? Isn't life sacred?
    - Is it discriminatory to stop life support treatment based on 'quality of life'?
    - Is quality of life a 'personal' decision?
    - Questions you could ask your doctor
- Uncertainty
    - Can the doctors be certain about what will happen for my child?
    - Questions you could ask your doctor about uncertainty
    - Ways to think about uncertainty
    - Is there a rush to make a decision? Do I need to decide now?
    - I have read about a new treatment/test on the internet…
- Who decides about life support?
    - Who makes the final decision about life support treatment?
    - What does shared decision-making mean?
    - Questions you might like to ask your doctor
    - Phrases that may be helpful
    - What if I don't want to decide?
    - Do parents need to consent to stopping treatment?
    - What would you do doctor?
- Disagreement
    - What should I do if the doctors don't agree about life support?
    - What should I do if I don't agree with my child's doctors about life support?
    - What should I do if my partner and I do not agree about life support treatment?
    - What should I do if others in my family do not agree about life support treatment?
- Religion, culture and life support
    - Is it against my religion to stop life support?
    - Is it playing God to stop life support?

    - What about the sanctity of life?
    - I don't want to stop life support because I am waiting for a miracle
    - Can I talk to my doctor about my cultural or religious beliefs?
    - Can I ask doctors to speak to my elder or religious advisor?
    - What do different religions say about stopping life support?
- After a decision
    - We have decided to continue life support. What will happen now?
    - We have decided to stop or not start life support. What will happen now?
    - What happens after a breathing tube is removed?
    - If my child has morphine, will it stop him/her breathing?
    - How long do we have?
    - What about organ or tissue donation?
    - What do I do about organising a funeral?
- Taboo questions
    - What if I had… If only I had…?
    - Is it bad to wish for my child to die?
    - Can't the doctors give my child something to put them out of their misery?
    - Do doctors want to stop life support for my child because it costs too much?
    - Is stopping life-support treatment giving up on my child?
    - Is there no hope?
    - The doctors cannot be right. They must have made a mistake.
    - If I agree to stop life support, will people think I am a bad parent?
    - Will we be abandoned?
    - Will I be letting the doctors down if I agree to stopping or not starting life support?
    - What about the rest of my family and their quality of life?
    - I am afraid that I won't be able to cope if…
    - 'Did I make the right decision?' Will I be able to live with myself?

- What to tell other people
    - Should I say anything?
    - What do I tell other people about life support?
    - What do I tell my other children?
- Further information
